# Genetically Low Vitamin D Levels, Bone Mineral Density, and Bone Metabolism Markers: a Mendelian Randomisation Study

**DOI:** 10.1038/srep33202

**Published:** 2016-09-14

**Authors:** Shan-Shan Li, Li-Hong Gao, Xiao-Ya Zhang, Jin-We He, Wen-Zhen Fu, Yu-Juan Liu, Yun-Qiu Hu, Zhen-Lin Zhang

**Affiliations:** 1 Metabolic Bone Disease and Genetics Research Unit, Department of Osteoporosis and Bone Diseases, Shanghai Jiao Tong University Affiliated Sixth People’s Hospital, Shanghai 200233, China; 2 Shanghai Key Clinical Center for Metabolic Disease, Shanghai 200233, China

## Abstract

Low serum 25-hydroxyvitamin D (25OHD) is associated with osteoporosis and osteoporotic fracture, but it remains uncertain whether these associations are causal. We conducted a Mendelian randomization (MR) study of 1,824 postmenopausal Chinese women to examine whether the detected associations between serum 25OHD and bone mineral density (BMD) and bone metabolism markers were causal. In observational analyses, total serum 25OHD was positively associated with BMD at lumbar spine (*P* = 0.003), femoral neck (*P* = 0.006) and total hip (*P* = 0.005), and was inversely associated with intact parathyroid hormone (PTH) (*P* = 8.18E-09) and procollagen type 1 N-terminal propeptide (P1NP) (*P* = 0.020). By contract, the associations of bioavailable and free 25OHD with all tested outcomes were negligible (all *P* > 0.05). The use of four single nucleotide polymorphisms, *GC*-rs2282679, *NADSYN1-*rs12785878*, CYP2R1-*rs10741657 and *CYP24A1-*rs6013897, as candidate instrumental variables in MR analyses showed that none of the two stage least squares models provided evidence for associations between serum 25OHD and either BMD or bone metabolism markers (all *P* > 0.05). We suggest that after controlling for unidentified confounding factors in MR analyses, the associations between genetically low serum 25OHD and BMD and bone metabolism markers are unlikely to be causal.

Vitamin D insufficiency is an increasingly prevalent public health issue worldwide and is regarded as one of the foremost modifiable risk factors for a several common diseases and conditions, including osteoporosis and osteoporotic fracture^1^,^2^,^3^. Serum 25-hydroxyvitamin D (25OHD) levels are the established clinical marker of vitamin D status. The findings from most observational studies have suggested associations between low serum 25OHD levels and secondary hyperparathyroidism, elevated levels of bone turnover markers and excessive bone loss^1^,^3^. Moreover, bone mineral density (BMD) and bone turnover markers are among the robust predictors of osteoporotic fracture risk^4^,^5^. However, meta-analyses of randomized controlled trials have provided little evidence that vitamin D supplements alone provide benefits of BMD improvement or fracture prevention^6^,^7^,^8^, thus suggesting vitamin D may not have a causal effect on bone health^9^,^10^. Notably, these findings require cautious interpretation. Observational investigations are susceptible to potential confounding factors and reverse causality^11^,^12^, whereas the dosage of vitamin D supplements, research duration, baseline vitamin D levels of the study population, and genetic factors inevitably affect the intervention response^7^,^8^,^13^. Therefore, whether these associations are causal remains uncertain.

An alternative approach to a causality study is a Mendelian randomization (MR) analysis, an increasingly used method that draws a causal inference from the observational data. MR analyses assess genetic variants predicted risk factors to screen their causal effects on the outcomes of interest^12^,^14^. Because the genetic variants are randomly assorted at conception, similarly to randomized trials, the MR approach is largely free of both residual confounding factors and reverse causation. Moreover, the MR approach has successfully clarified the causal effect of low 25OHD levels on the increased risk of type 2 diabetes^15^ and of high urate levels on a reduced rate of Parkinson disease progression^16^, and has confirmed that genetically low urate levels and decreased BMD are not causally related^17^. Our previous study of 2,897 healthy Chinese subjects has suggested that the *GC*, *CYP2R1* and *NADSYN1* polymorphisms within the vitamin D metabolic pathway are genetic determinants of variations in serum 25OHD levels^18^, and these findings have provided a basis for our present MR analyses in a Chinese population.

In this study, we sought to verify whether serum 25OHD levels had a strong causal effect on bone health by using the MR approach. First, we examined the observational associations between the total serum 25OHD and BMD and bone metabolism markers [i.e., intact parathyroid hormone (PTH), Beta-CrossLaps of type I collagen containing cross-linked C-telopeptide (Beta-CTX) and procollagen type 1 N-terminal propeptide (P1NP)] using ordinary least squares (OLS) models. Second, we calculated the free and bioavailable (free + albumin bound) 25OHD levels by using Vermeulen formulas based on directly measured values of the total serum 25OHD, vitamin D binding protein (DBP) and albumin levels^19^, and determined whether the bioavailable and free 25OHD levels were more closely associated with BMD and bone metabolism markers. Then, we determined whether the verified observational associations were causal by using different two stage least squares (TSLS) models based on the assumptions of the MR analyses^12^,^14^,^20^.

## Results

### General characteristics of the study population

Of the 2,013 participants, 158 subjects (7.9%) were excluded because they had diseases or took medications that might affect bone or vitamin D metabolism. We excluded an additional 31 subjects (1.5%) with abnormal plasma glucose, serum calcium, or phosphorus levels and those with abnormal renal or liver function. 1,824 participants were included in our study, and their general characteristics are summarized in Table 1. The average age was 65.5 (SD 8.9) years, the average BMI was 23.5 (SD 3.3) kg/m^2^, and the median (25^th^ and 75^th^ percentiles) total serum 25OHD level was 18.3 (13.3, 23.8) ng/mL.

### Observational relationships between serum 25OHD and clinical traits

OLS regression analyses provided strong evidence of observational associations between the total serum 25OHD levels and BMD at different sites (i.e., lumbar spine at L1-L4, femoral neck, and total hip); these associations remained robust after adjusting for age, season and BMI (Table 2). The absolute changes in BMD at L1-L4, femoral neck, and total hip were 0.047 g/cm^2^ (*P* = 0.003), 0.031 g/cm^2^ (*P* = 0.006) and 0.034 g/cm^2^ (*P* = 0.005) per unit increase in adjusted total serum 25OHD, respectively. Subjects with lower total 25OHD levels were shown to have significantly higher PTH values (Beta = −0.103, *P* = 8.18E-09) and P1NP values (Beta = −0.088, *P* = 0.020) independent of age, season and BMI. However, no significant associations were observed between the serum levels of bioavailable or free 25OHD and BMD, PTH, Beta-CTX or P1NP (all *P* > 0.05) after controlling for age, season, and BMI in the OLS regression analyses (Supplementary Table 1). We conducted ANOVA tests for normal data and Kruskal-Wallis tests for non-normal data in the subsequent analyses to address possible bias resulting from the multicollinearity of the present analyses. However, we still did not identify any significant associations between the serum levels of bioavailable or free 25OHD and the tested outcomes (Supplementary Tables 2 and 3).

### Associations between SNPs with total serum 25OHD levels, clinical traits and potential confounding factors

Table 3 presents the basic information pertaining to the SNPs. The minor allele frequencies (MAFs) of the 10 SNPs selected in the present study were similar to the HapMap-CHB reference data, and none of the SNPs failed the quality control checks. Four SNPs (*GC*-rs2282679, *NADSYN1-*rs12785878*, CYP2R1-*rs10741657 and *CYP24A1-*rs6013897) were considered as candidate instrumental variables (IVs) by default on the basis of their repeatable genome-wide significant associations with the 25OHD levels from genome-wide association study (GWAS) data^21^. Because high linkage disequilibrium was identified in our study between *GC*-rs4588, *NADSYN1-*rs2276360 and *CYP2R1*-rs2060793 and candidate IVs *GC*-rs2282679 (r^2^ = 0.95), *NADSYN1-*rs12785878 (r^2^ = 0.99) and *CYP2R1-*rs10741657 (r^2^ = 0.99), respectively, we selected the last 3 SNPs as proxies in the subsequent MR analyses. For the remaining 3 SNPs, the minor alleles of both *GC*-rs1155563 and *CYP2R1-*rs10766197 were significantly related to decreased total serum 25OHD levels after adjusting for age, BMI and season of blood draw, but these fell below the significance threshold after False Discovery Rate (FDR) correction (Table 4). Therefore, these 3 SNPs were excluded from the subsequent analyses. We then examined whether the 4 selected IVs were associated with tested outcomes (i.e., BMD, PTH and P1NP) or potential confounding factors (i.e., age, BMI, Ca, P, Cr and BUN), and no significant evidence of associations was identified after FDR correction (Supplementary Tables 4 and 5). Therefore, 4 SNPs, including rs2282679, rs12785878, rs10741657 and rs6013897, were considered to be the genetic IVs in the subsequent MR analyses.

### Evaluation of causal associations between 25OHD levels and clinical outcomes

We used both single instrument model and multiple instruments model in the present study. According to previous GWAS data^21^, *GC*-rs2282679 exhibited the strongest association with the 25OHD levels and was used as the IV in the single instrument model. The multiple instruments models, including unweighted allele scores model and weighted allele scores model, were performed on the basis of the sum of the number of effect alleles of the 4 candidate IVs. The variability in the log-transformed total serum 25OHD levels explained by each IV ranged from 0.7% to 1.1% (Table 5). The lowest relative TSLS/OLS bias ratio was observed in both the single instrument model and the weighted allele scores model. The *F*-statistic values, which indicated the instrument strength of the MR analyses, are shown in Table 5. As a rule of thumb, all 3 MR analyses models were considered to have strong instruments for the *F*-statistic values obtained from first stage regression analyses with values more than 10; thus, all 3 MR models were used to assess the causal association.

As presented in Table 6, using the SNP (rs2282679) for the single instrument analyses, we found no significant evidence of a causal effect of the total serum 25OHD levels on BMD, PTH or P1NP (all *P* > 0.05), with SE values ranging from 0.056 to 0.139. The Hausman Test indicated significant differences in the IV estimates compared with the OLS estimates of the effect of the 25OHD levels on the 4 tested outcomes after FDR correction (all *P* < 0.05). We then performed MR analyses based on the unweighted and weighted allele scores models, and the IV estimates from the weighted allele scores models were similar to the estimates from the OLS models and single instrument models. Notably, the inclusion of the weighted allele score as a genetic instrument in the MR analyses led to a considerable decrease in the SE values compared with those of the single instrument model, which corresponded to considerably increased statistical power^14^. However, using multiple instruments models described above, we still were unable to verify that the total serum 25OHD levels were causally associated with either BMD or bone metabolism markers. Consistently with the results from the single instrument models, the estimates of the effect of the total serum 25OHD levels on BMD at all sites and PTH from both the unweighted allele scores models and the weighted allele scores models were significantly different from the estimates from OLS models even after FDR correction (all *P* < 0.05).

## Discussion

In this study of 1,824 postmenopausal Chinese women, our three key findings were as follows: 1) the total serum 25OHD levels were positively associated with the BMD at L1-L4, femoral neck, and total hip and were inversely associated with the serum PTH and P1NP levels; 2) the serum levels of bioavailable or free 25OHD were not associated with any of the tested BMD sites or bone metabolism markers; and 3) the MR analyses showed that genetically low serum 25OHD levels were not associated with decreased BMD or with elevated serum PTH or P1NP levels. These results suggested that the total serum 25OHD levels were unlikely to have a robust causal effect on BMD or bone metabolism markers, but could serve as a marker thereof.

Our observational analyses showed strong associations between the total serum 25OHD levels and BMD, serum PTH and P1NP; these associations were independent of age, season and BMI. These results were consistent with the results of several^22^,^23^, but not all^24^,^25^,^26^, previous studies. A significant relationship between the 25OHD levels and the total hip BMD has also been observed in the NHANESIII study, which included 13,432 subjects^22^. Saliba and colleagues have revealed an inverse correlation between the serum 25OHD levels and the serum PTH levels^23^. Moreover, the Peking Vertebral Fracture study conducted by Zhao *et al*. has shown that serum 25OHD levels are negatively correlated with P1NP levels in postmenopausal Chinese women, but the authors were unable to identify any associations between the 25OHD levels and BMD at any sites^24^. Garnero *et al*. have observed only a modest correlation between the serum 25OHD levels and intact PTH levels, but not the total hip BMD or bone turnover markers, in home-dwelling, healthy postmenopausal women^25^. Additionally, Nimitphong *et al*. have found that only subjects with certain DBP genotypes show a positive association between serum 25OHD levels with BMD^26^. According to the free hormone hypothesis, which states that only hormones released from binding proteins (i.e., DBP) are able to act on target cells to exert biological effect^27^, the bioavailable and free 25OHD levels are thought to represent the serum 25OHD levels that are available for biological activity. This hypothesis might partially explain the conflicting findings drawn from different association analyses of the total serum 25OHD levels and bone health. In a cross-sectional study including 49 healthy, young subjects, Powe and colleagues have established a closer relationship between the free and bioavailable 25OHD levels and BMD than between the total 25OHD levels and BMD^28^. In addition, the finding from a study of 265 postmenopausal women has also indicated that the serum levels of free and bioavailable 25OHD might provide more information about vitamin D status in relation to BMD^29^. In a community-based study of African and Caucasian Americans, both groups have been found to show similar PTH levels, owing to their similar levels of bioavailable 25OHD, though African Americans commonly have lower total 25OHD levels^30^. These studies suggest that, like other serum hormone carrier proteins, circulating DBP might play an important regulatory role in the biologic action of human vitamin D. However, Dastani and colleagues have discovered that the biological effect of vitamin D on PTH levels is mainly independent of DBP in a large vitamin D-sufficient cohort^31^. Moreover, an 8-week randomized controlled trial has also indicated that for individuals with total 25OHD levels <20 ng/mL, the serum DBP levels do not influence the effect of vitamin D supplements on either serum PTH or calcium levels^32^. Although the levels of bioavailable 25OHD in both the treatment and placebo groups showed significant associations with the PTH levels, these relationships were weaker than the relationship between PTH and total 25OHD^32^. However, in our study, we did not identify any significant observational associations between bioavailable or free 25OHD and either BMD or bone metabolism markers. These conflicting findings might be due to the different study populations, which had various ages, genders, genetic differences, and vitamin D nutrition. Our study enrolled postmenopausal women, whereas Powe *et al*. studied young males and females^28^. The study population recruited by Johnsen *et al*. was limited to postmenopausal women with low BMD, and the significant associations between the 25OHD levels and BMD were not fully consistent at all sites^29^. Thus, further investigation in a larger study population may be required. Additionally, different DBP kits might also lead to different results. The DBP kit used in our study was identical to the kit used by most of the studies mentioned above^28^,^29^,^32^. In addition, it has been confirmed that the serum levels of bioavailable and free 25OHD calculated using the estimated formulas are highly consistent with the measured values^30^. Therefore, the differences in the findings might not primarily result from the DBP detection method or the formulas used to calculate the 25OHD levels. Alternatively, it was possible that the nominally significant relationships between total serum 25OHD and either BMD or bone metabolism markers might largely be ascribed to some unidentified confounding factors in the observational analyses. Moreover, the serum 25OHD levels might have only mild associations with BMD and with bone metabolism markers in postmenopausal Chinese women^25^.

Our MR analyses provided no evidence of a causal role of genetically low serum 25OHD levels in either decreased BMD or elevated serum PTH or P1NP, though our study might not have had sufficient power to detect very small effects. The Hausman Test revealed significant differences between the OLS estimates and TSLS estimates of the 25OHD levels in relation to BMD and PTH in the three different MR models, thus strongly suggesting the existence of unmeasured confounding factors in the observational analyses and providing further support for the conclusion drawn from the MR analyses^12^,^20^. Our negative results for a causal effect of the serum 25OHD levels on BMD were in accord with the findings derived from meta-analyses of the validity of using vitamin D supplements alone to improve bone health^6^,^8^. Reid and co-workers have revealed only a small benefit at the femoral neck from vitamin D supplements, with heterogeneity among trials, and have ascribed this localized effect to chance after ruling out the possibility of a cortical-specific effect^8^. Furthermore, the pooled data from vitamin D fracture trials in the US and Europe have indicated that the administration of a daily dose of 400–800 IU vitamin D alone is ineffective at preventing fracture^6^. Our present findings, as well as the conclusions drawn from the intervention trials described above, indicated the possibility of a lack of causal evidence showing that vitamin D alone can improve BMD or prevent fracture. According to previous studies, the maximum suppression of serum PTH has been used to define an optimal serum 25OHD levels^33^,^34^. In addition, it has been verified that a persistent 25OHD deficiency would maximally stimulate the parathyroid glands and thus lead to secondary hyperparathyroidism, but two-thirds of study participants lacked relevant changes^35^. The precise mechanism underlying the seemingly impaired PTH response is not readily apparent. PTH is among the major hormones responsible for serum calcium and phosphorus homeostasis^1^,^3^. Parathyroid glands are abundant in vitamin D receptors (VDRs) and are thus established as target tissues for vitamin D action^36^. 1,25-dihydroxyvitamin D [1,25(OH)_2_D], which is derived from 25OHD by 1-hydroxylation in the kidney, is the metabolically active form on behalf of vitamin D activity^37^. The inhibitory effect of 1,25(OH)_2_D on PTH gene transcription and parathyroid cell hyperplasia has been clarified both *in vitro* and *in vivo* studies^36^,^38^. Moreover, it has been shown that decreased calcium absorption, which accounts for elevated serum PTH levels, is precisely regulated by 1,25(OH)_2_D^37^,^39^. Notably, serum 25OHD levels do not exhibit a direct causal effect on calcium absorption, but might exert a permissive action for regulating absorption through 1,25(OH)_2_D, thus regulating the PTH levels^34^,^37^. These findings may provide possible explanation for the lack of a causal association between serum 25OHD and PTH in the MR analyses.

To our knowledge, this is the first study to investigate the causal relationships between the serum 25OHD and BMD and bone metabolism markers by using the MR approach while gaining insights into the associations of bioavailable and free 25OHD with the clinical outcomes listed above. The MR analyses assessed the effects of the genetic variants predicted lifelong low serum 25OHD levels on the clinical outcomes of interest. Because the genetic variants were randomly distributed at meiosis and remained unchanged throughout life, the MR analyses were free of confounding factors and reverse causality, thus strengthening the causal conclusions. We included tagging SNPs as potential genetic IVs in this study, thus maximizing the amount of 25OHD variability explained by each SNP^11^,^14^ and increasing the statistical power to evaluate the causal associations between genetically low serum 25OHD levels and the clinical outcomes. Because the estimates obtained from the TSLS models and the OLS models were comparable, there was little possibility of violating the assumptions of the MR analyses^12^. Notably, among the TSLS models, the weighted allele scores model using external weights for multiple genetic variants showed the lowest SE values and corresponded to the greatest power. Although no recognized formulas for calculating sample sizes were available for MR analyses using multiple genetic variants, the results showed that a 20% decrease in SE would lead to a 56% increase in the practicable sample size^14^. However, we acknowledge that the study has several potential limitations. Although the lack of causal associations between the serum 25OHD levels and the tested clinical outcomes were confirmed in three different MR analyses models, the models had a limited ability to exclude very small effects. In addition, it was possible that the null findings were caused by biological adaptations of the genetic variants; in other words, the phenotypic influence of these SNPs might have been buffered during development^40^. An MR analysis depends on the assumptions that the genetic variants are specially associated with the risk factor (i.e., the serum 25OHD levels) and then act on the outcomes. However, a violation of these assumptions occurs when the genetic variants are pleiotropic or are in linkage disequilibrium with unknown functional variants related to the tested outcomes^12^,^14^. In addition, because the MR analyses were based on assumed linear relationships between the intermediate variables and the outcomes, it was not possible to estimate the different causal effects arising from the different physiological ranges of the serum 25OHD levels. Because the association analyses for the serum levels of bioavailable and free 25OHD were based on values calculated with established formulas, a confirmation of our findings using directly measured values would be ideal. Finally, our study population was limited to postmenopausal Chinese women, and the findings drawn from this study might not be appropriate for other population groups of different ages, sexes, or ethnic backgrounds.

In conclusion, our study affirmed the observational associations between the total serum 25OHD levels with BMD and serum PTH and P1NP levels, but provided no evidence of relationships between the serum levels of bioavailable or free 25OHD and the above clinical traits. The MR analyses indicated the existence of unrecognized confounding factors in the observational analyses and showed that genetically low serum 25OHD levels were not casually associated with any of the tested outcomes in postmenopausal Chinese women. We suggest that vitamin D, like other micronutrients, is necessary for and serve as a good marker of bone health, but it might not be a direct causative factor.

## Methods

### Study subjects

This cross-sectional study involved 2,013 genetically unrelated Han Chinese women who had been postmenopausal for more than 1 year, were living in Shanghai, and were recruited from the Department of Osteoporosis and Bone Diseases Outpatient Clinic of Shanghai Jiao Tong University Affiliated Sixth People’s Hospital. A standardized questionnaire was used to collect information about the date of birth, age at menarche and amenorrhea, life style, medical history and medication use. Participants with any disease or medication treatment known to affect bone or vitamin D metabolism were excluded. The selected subjects then attended a medical examination and provided blood after overnight fasting to determine the blood counts, fasting plasma glucose levels, serum calcium levels, serum phosphorus levels, serum albumin levels, and liver and renal function. Subjects with normal results for the physical and biochemical examinations were considered eligible for our study. The study was approved by the Ethics Committee of the Shanghai Jiao Tong University Affiliated Sixth People’s Hospital, and written informed consent was obtained from each participant. All experiments were performed in accordance with the approved guidelines and regulations.

### BMD measurements

BMD values (g/cm^2^) of the spine at L1-L4, the left femoral neck, and the total hip were measured using dual-energy X-ray absorptiometry (DXA) on a Lunar Prodigy GE densitometer (Lunar Corp, Madison, WI, USA). Subjects with a history of left femur fracture or surgery received a right femoral neck measurement instead. We calibrated the machine daily through triplicate measurements of the same 15 individuals; the coefficient of variability (CV) values of the BMD at L1-L4, femoral neck, and total hip were 1.39%, 2.22% and 0.70%, respectively^41^. The long-term reproducibility of the DXA data during the study was 99.55%, which was determined by weekly repeated phantom measurements. BMI (kg/m^2^) was calculated as the weight divided by the height squared. All measurements were conducted by the same well-trained technicians throughout the study.

### Biochemical assays

Serum samples were collected between 08:00 and 10:00 AM after overnight fasting of at least 12 hours and then stored at −80 °C. The total serum 25OHD and bone metabolism markers (i.e., intact PTH, Beta-CTX and P1NP) levels were measured using an automated Roche electro-chemiluminescence system (E170; Roche Diagnostic GmbH, Mannheim, Germany) according to the manufacturer’s protocol and specialized assay laboratory quality control procedures. The intra- and interassay CVs were 5.7% and 7.3% for 25OHD, 1.4% and 2.9% for PTH, 2.5% and 3.5% for Beta-CTX, 2.9% and 3.8% for P1NP, respectively^41^.

### Calculation of the serum levels of bioavailable and free 25OHD

The serum DBP levels were determined using a commercial enzyme-linked immunosorbent assay (Catalogue Number DVDBP0, R&D Systems) according to the manufacturer’s instructions. The intraassay CV was 5–7% and the interassay CV was 5–8%. The detectable range could be expanded with an appropriate sample dilution. The bioavailable (non-DBP fraction) and free 25OHD levels were calculated by using Vermeulen equations^20^ on the basis of the measured total serum 25OHD, DBP, and albumin levels and the respective binding affinity constants between DBP, albumin, and 25OHD. The calculated serum 25OHD levels were validated to correlate well with the values that were directly measured with a competitive radioligand binding assay in several previous studies^28^,^30^,^31^.

### SNP selection and genotyping

On the basis on 25OHD GWAS data and previous association studies in Chinese populations^18^,^21^,^42^, we selected 10 SNPs within the vitamin D metabolic pathway (i.e., *GC*-rs4588, *GC*-rs7041, *GC*-rs2282679, *GC*-rs1155563, *NADSYN1*-rs2276360, *NADSYN1-*rs12785878*, CYP2R1*-rs2060793, *CYP2R1-*rs10741657*, CYP2R1-*rs10766197 and *CYP24A1-*rs6013897) as potential IVs for the MR analyses. Blood samples were collected from all of the participants, and genomic DNA was extracted and purified from peripheral blood leukocytes by using a QuickGene DNA whole blood kit L by Nucleic Acid Isolation System (QuickGene-610L, FUJI FILM, Japan). Genotyping was performed with an ABI PRISM SNaPshot multiplex kit (Applied Biosystems), an Mx3000p real-time PCR system (Stratagene), and GeneMapper 4.0 (Applied Biosystems). Genotype frequencies were estimated on the basis of Hardy-Weinberg Equilibrium (HWE) with a chi-square test to detect genotyping errors.

### Statistical analyses

We assessed the distribution of all continuous variables and excluded the extreme values [>3.5 standard deviations (SD) from the mean; <1% of all data points]. Descriptive statistics were reported as the means ± SD for normally distributed data and as medians (25^th^ and 75^th^ percentiles) for non-normally distributed data. Natural logarithmic transformation was used for the skewed variables to approximate normality for the subsequent data analyses. The statistical analyses were performed using SPSS version 13.0 (SPSS Inc., Chicago, IL, USA), PLINK (http://pngu.mgh.harvard.edu/purcell/plink/) was used for SNP quality control filtering and association tests, and the linkage disequilibrium analyses among selected SNPs were conducted with Haploview 4.2 (http://www.broadinstitute.org/scientific-community/science/programs/medical-and-population-genetics/haploview/haploview). The null hypothesis was tested using alpha = 0.05 (two-sided). The FDR method was applied to control the family-wise error rate when multiple tests were performed.

**Figure 1:**
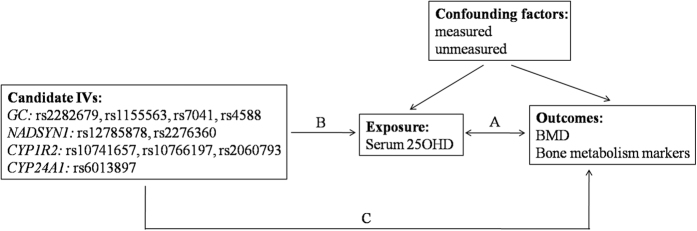
Framework of the Mendelian randomization analysis used in this study. IVs, instrumental variables; BMD, bone mineral density. (**A**) indicates an observational association between the exposure and the outcomes; (**B**) indicates the direction of the associations of the genetic variants with the exposure; (**C**) indicates the direction of the genetically determined exposure with the outcomes.

First, OLS regression models were performed to examine the observational associations between the serum levels of total, bioavailable and free 25OHD with the tested clinical traits after adjusting for age, BMI and season, and to obtain the estimates of the effect of the serum 25OHD levels on the tested variables in each model (Fig. 1A). Because of the possibility of existence of multicollinearity in linear regression models that may have obscured the significant associations, we divided the data into four groups according to the quartiles of the serum 25OHD levels and assessed the differences in the clinical variables with an ANOVA test or a Kruskal-Wallis test, as appropriate. The verified observational associations were further analysed using TSLS models to determine whether the genetically predicted 25OHD levels were casually associated with the tested clinical traits.

Second, as components of the MR analyses, we examined the following IV assumptions^14^: 1) the genetic variants were associated with exposure; 2) the genetic variants were independent of unmeasured confounding factors; and 3) the genetic variants were related to the outcomes only via their associations with the intermediate phenotype (i.e., 25OHD levels) (Fig. 1B). SNPs that passed the quality control checks (*P* > 0.05 for HWE test; genotyping rate >90%) were included in the subsequent analyses. The first-stage regression *F*-statistic and coefficient of determination, *R*^*2*^, were used to assess the strength of the SNPs as possible IVs. The relative bias of the TSLS regression estimator compared with the OLS regression estimator was calculated by using verified equations based on the *F*-statistic values^14^. We performed linear regression analyses to evaluate the associations of the potential IVs with the underlying confounding factors (i.e., age, BMI, Ca, P, Cr and BUN) and to exclude the direct associations of the IVs with the clinical outcomes (i.e., BMD and bone metabolism markers).

Third, we constructed different MR models based on selected IVs, including single instrument model, unweighted allele scores model, and weighted allele scores model, to examine the effects of the different genetic markers determined serum 25OHD levels on the tested traits (Fig. 1C). These models were intended to provide a more promising method for our causal studies while addressing possible issues arising from the violated IV assumptions. In the MR analyses, genotypes were coded as 0, 1, and 2 across the number of effect alleles, namely vitamin D lowering alleles. The allele score was calculated by counting the number of candidate IVs effect alleles. The analyses of the associations of the genetically determined serum 25OHD levels with the tested outcomes were estimated by using two stage regression models. The first stage generated the genetically predicted 25OHD values by using a linear regression model of the adjusted 25OHD levels, based on the estimates for the genetic IVs. The second stage was performed in a multivariable linear regression model of the clinical outcomes versus the predicted 25OHD values after controlling for the age, BMI, season, and estimated residuals. For the weighted allele scores model, the weights were external to our present study and were taken from the overall evidence from previous studies to decrease possible bias^11^,^42^. The Hausman Test was used to check for the endogeneity by comparing the TSLS estimates with the OLS estimates^14^.

## Additional Information

**How to cite this article**: Li, S.-S. *et al*. Genetically Low Vitamin D Levels, Bone Mineral Density, and Bone Metabolism Markers: a Mendelian Randomisation Study. *Sci. Rep.* **6**, 33202; doi: 10.1038/srep33202 (2016).

## Supplementary Material

Supplementary Information

## Figures and Tables

**Table 1:** Baseline characteristics of the study participants.

Characteristics	Total sample (n = 1824)	Minimum	Maximum
Age (years)	65.5 ± 8.9	43.1	94.9
Height (cm)	154.2 ± 6.0	133.0	175.0
Weight (kg)	55.8 ± 8.4	29.0	81.0
BMI (kg/m^2^)	23.5 ± 3.3	14.2	36.5
Total 25OHD (ng/mL)	18.3 (13.3–23.8)	4.0	57.4
Vitamin D binding protein (mg/L)	152.9 (100.7–240.8)	43.7	482.9
Albumin (g/L)	46.0 (44.0–48.0)	21.0	55.0
Bioavailable 25OHD (ng/mL)	3.7 (2.4–5.5)	0.4	16.3
Free 25OHD (pg/mL)	8.8 (5.9–13.5)	1.1	38.9
PTH (pg/mL)	40.7 (32.2–52.1)	17.4	100.3
P1NP (g/L)	57.0 (43.5–73.7)	16.4	143.0
Beta-CTX (ng/L)	392.5 (282.0–533.0)	88.0	1030.0
ALP (U/L)	72.9 ± 16.8	23.0	112.0
Ca (mmol/L)	2.33 ± 0.10	2.08	2.60
P (mmol/L)	1.16 ± 0.14	0.80	1.60
Cr (μmol/L)	58.9 ± 10.6	34.0	102.0
BUN (mmol/L)	5.1 ± 1.3	1.9	10.2
Lumbar 1–4 BMD (g/cm^2^)	0.877 ± 0.141	0.419	1.695
Femoral neck BMD (g/cm^2^)	0.722 ± 0.110	0.362	1.301
Total hip BMD (g/cm^2^)	0.765 ± 0.118	0.321	1.260

Normally distributed variables are presented as the means ± standard deviation, and non-normally distributed variables are presented as medians (interquartile range).

**Table 2:** Observational associations between the total serum 25OHD levels and the clinical variables.

Varibales	Effect estimates^a^	*P* Value^a^	Effect estimates^b^	*P* Value^b^
Lumbar 1–4 BMD (g/cm^2^)	0.043	**0.008**	0.047	**0.003**
Femoral neck BMD (g/cm^2^)	0.036	**0.004**	0.031	**0.006**
Total hip BMD (g/cm^2^)	0.039	**0.004**	0.034	**0.005**
PTH (pg/mL)	−0.114	**2.302E-11**	−0.103	**8.181E-09**
Beta-CTX (ng/L)	−0.029	0.350	−0.023	0.483
P1NP (g/L)	−0.082	**0.029**	−0.088	**0.020**

The False Discovery Rate (FDR) method was used to control the family-wise error rate when multiple hypotheses tests were performed. The null hypothesis was tested using alpha = 0.05 (two-sided). Significant values are presented in bold. Serum PTH, Beta-CTX, P1NP, and 25OHD levels were log-transformed to approximate normality.

^a^Effect estimates are presented as changes in the clinical variables per unit increase in the log-transformed serum 25OHD levels.

^b^Adjusted for age, season and BMI.

**Table 3:** 25OHD single nucleotide polymorphisms (SNPs) selected for this study.

Chromosome	Gene (SNP)	SNP Property	Allele	Functional Change	MAF	HWE test *P* Value
4	*GC* (rs4588)	Exon 11	G/T	p.Thr436Lys	0.333	0.755
4	*GC* (rs7041)	Exon 11	A/C	p.Asp432Glu	0.253	0.805
4	*GC* (rs2282679)	Intron 12	T/G	NA	0.338	0.747
4	*GC* (rs1155563)	Intron 1	T/C	NA	0.409	0.683
11	*NADSYN1* (rs2276360)	Exon 3	G/C	p.Val74Leu	0.461	0.120
11	*NADSYN1*(rs12785878)	intron2	G/T	NA	0.462	0.177
11	*CYP2R1* (rs2060793)	5′-flanking	G/A	NA	0.379	1.000
11	*CYP2R1*(rs10741657)	5′-flanking	G/A	NA	0.379	0.808
11	*CYP2R1* (rs10766197)	5′-flanking	G/A	NA	0.345	0.573
20	*CYP24A1* (rs6013897)	3′-flanking	T/A	NA	0.162	0.611

SNP, single nucleotide polymorphism; Allele, major allele/minor allele; NA, not available; MAF, minor allele frequency; HWE, Hardy-Weinberg Equilibrium.

**Table 4:** Effect of the genetic polymorphisms on the total serum 25OHD levels.

SNP	Beta	SE	95%CI		*P* Value
			lower limit	upper limit	
rs7041	0.010	0.009	−0.007	0.027	0.229
rs1155563	−0.017	0.008	−0.031	−0.002	0.024
rs10766197	−0.016	0.008	−0.032	−0.001	0.039

SNP, single nucleotide polymorphism; Beta, regression coefficient; SE, standard error; CI, confidence interval. The False Discovery Rate (FDR) method was used to control the family-wise error rate when multiple hypotheses tests were performed. The null hypothesis was tested using alpha = 0.05 (two-sided). Significant values obtained after FDR correction are presented in bold. The analyses were performed under additive models by adjusting for age, season and BMI. The total serum 25OHD levels were log-transformed to approximate normality. Beta refers to the changes in the log-transformed total serum 25OHD levels per each additional copy of the minor allele.

**Table 5:** Instrument strength and relative sample bias of the two stage least squares (TSLS) models on the total serum 25OHD levels.

Models	*R* ^*2*^	*F*-statistic	Relative TSLS/OLS bias ratio
Single instrument model			
rs2282679	0.011	20.26	0.050
Multiple instruments model			
Unweighted allele scores	0.007	12.84	0.078
Weighted allele scores	0.011	20.26	0.050

OLS, ordinary least squares. The multiple instruments model was based on the sum of the number of effect alleles of rs2282679, rs12785878, rs10741657, and rs6013897. The serum 25OHD levels were log-transformed to approximate normality.

**Table 6:** Two stage least squares (TSLS) estimates of the effects of the total serum 25OHD levels on bone mineral density (BMD) and bone metabolism markers.

Variables	Methods	Beta	SE	*P* Value	Hausman Test *P* Value
Lumar 1–4 BMD (g/cm^2^)	Model 1	−0.087	0.080	0.282	**0.002**
	Model 2	−0.137	0.190	0.472	**0.002**
	Model 3	−0.048	0.056	0.384	**0.002**
Femoral neck BMD (g/cm^2^)	Model 1	−0.040	0.056	0.477	**0.005**
	Model 2	−0.138	0.133	0.299	**0.006**
	Model 3	−0.044	0.039	0.261	**0.005**
Total hip BMD (g/cm^2^)	Model 1	−0.017	0.060	0.784	**0.004**
	Model 2	−0.130	0.143	0.364	**0.004**
	Model 3	−0.041	0.042	0.326	**0.004**
PTH (pg/mL)	Model 1	−0.045	0.089	0.617	**2.780E-08**
	Model 2	0.463	0.211	0.028	**7.888E-09**
	Model 3	0.088	0.062	0.152	**8.912E-09**
P1NP (g/L)	Model 1	−0.108	0.139	0.440	0.046
	Model 2	−0.385	0.337	0.254	0.046
	Model 3	−0.099	0.098	0.312	0.051

Beta, regression coefficient; SE, standard error; Model 1, single instrument model; Model 2, unweighted allele scores model; Model 3, weighted allele scores model. The False Discovery Rate (FDR) method was used to control the family-wise error rate when multiple hypotheses tests were performed. The null hypothesis was tested using alpha = 0.05 (two-sided). Significant values are presented in bold. The serum PTH and P1NP levels were log-transformed to approximate normality. Beta refers to the changes in bone mineral density or bone metabolism markers per 1 unit increases in the log-transformed serum 25OHD levels, and the analyses are adjusted for age, season and BMI. The Hausman Test was used to check for the endogeneity by comparing the TSLS estimates with the OLS estimates.
