# Supplementary material for: Genetically Low Vitamin D Levels, Bone Mineral Density, and Bone Metabolism Markers: a Mendelian Randomisation Study
Source: Sci Rep. 2016 Sep 14;6:33202. doi: 10.1038/srep33202 (PMC5021966; doi:10.1038/srep33202)
Supplement: Supplementary Information [file srep33202-s1.pdf]

Genetically Low Vitamin D Levels, Bone Mineral Density, and Bone Metabolism Markers: a Mendelian Randomisation Study

Shan-Shan Li, Li-Hong Gao, Xiao-Ya Zhang, Jin-Wei He, Wen-Zhen Fu, Yu-Juan Liu, Yun-Qiu Hu, Zhen-Lin Zhang*

	**Bioavailable 25OHD (ng/mL)**				**Free 25OHD (pg/mL)**			
**Varibales**	**Effect estimates[a]**	***P* Value[a]**	**Effect estimates[b]**	***P* Value[b]**	**Effect estimates[a]**	***P* Value[a]**	**Effect estimates[b]**	***P* Value[b]**
Lumbar 1-4 BMD (g/cm^2)	0.015	0.386	0.008	0.637	0.012	0.510	0.005	0.755
Femoral neck BMD (g/cm^2)	0.011	0.429	-0.001	0.960	0.004	0.769	-0.003	0.838
Total hip BMD (g/cm^2)	0.006	0.704	-0.007	0.610	-0.003	0.864	-0.011	0.392
PTH (pg/mL)	-0.028	0.160	-0.025	0.201	-0.037	0.071	-0.036	0.071
Beta-CTX (ng/L)	0.022	0.404	0.022	0.405	0.020	0.462	0.021	0.421
P1NP (g/L)	-0.013	0.543	-0.015	0.476	-0.018	0.402	-0.019	0.385

Supplementary Table 1. Observational associations among the serum levels of bioavailable and free 25OHD with the clinical variables. The False Discovery Rate (FDR) method was used to control the family-wise error rate when multiple hypotheses tests were performed. The null hypothesis was tested using alpha = 0.05 (two-sided). Significant values are presented in bold. The serum PTH, Beta-CTX, P1NP, and 25OHD levels were log-transformed to approximate normality. [a]Effect estimates are presented as changes in the clinical variables per unit increase in the log-transformed serum 25OHD levels. [b]Adjusted for age, season and BMI.

Varibales	Bioavailable 25OHD (ng/mL)				
	**Group 1<br>< 2.4**	**Group2<br>2.4-3.7**	**Group3<br>3.7-5.5**	**Group4<br>> 5.5**	***P* Value[a]**
n	248	283	268	251	
Lumbar 1-4 BMD (g/cm^2)	0.884 ± 0.140	0.889 ± 0.136	0.894 ± 0.140	0.889 ± 0.141	0.905
Femoral neck BMD (g/cm^2)	0.720 ± 0.115	0.734 ± 0.116	0.731 ± 0.111	0.725 ± 0.099	0.880
Total hip BMD (g/cm^2)	0.766 ± 0.126	0.778 ± 0.120	0.775 ± 0.122	0.767 ± 0.105	0.625
PTH (pg/mL)	42.1 (32.0 - 52.6)	40.4 (31.6 - 52.6)	39.0 (31.3 - 52.0)	39.7 (30.8 - 49.8)	0.428
Beta-CTX (ng/L)	383.5 (272.3 - 491.0)	404.0 (305.0 - 541.0)	395.0(283.0 - 540.0)	388.0 (276.0 - 545.0)	0.141
P1NP (g/L)	56.0 (43.0 - 71.2)	59.2 (45.4 - 75.3)	57.9 (44.6 - 75.7)	55.0 (41.3 - 72.5)	0.174

Supplementary Table 2. Differences in the clinical variables according to the serum levels of bioavailable 25OHD. Normally distributed variables are presented as means ± standard deviation, and non-normally distributed variables are presented as medians (interquartile range). The null hypothesis was tested using alpha = 0.05 (two-sided). ANOVA tests were used for normal data, and Kruskal-Wallis tests were used for non-normal data when multiple group comparisons were performed. [a]Adjusted for age, season and BMI.

Varibales	Free 25OHD (pg/mL)				
	**Group1 < 5.5**	**Group2 5.9-8.8**	**Group3 8.8-13.5**	**Group4 > 13.5**	***P* Value**[a]
n	266	257	268	259	
Lumbar 1-4 BMD (g/cm^2)	0.889 ±0.140	0.883 ±0.138	0.896 ±0.139	0.888 ±0.139	0.596
Femoral neck BMD (g/cm^2)	0.726 ±0.114	0.726 ±0.119	0.739 ±0.111	0.720 ±0.097	0.533
Total hip BMD (g/cm^2)	0.772 ±0.123	0.770 ±0.123	0.783 ±0.121	0.761 ±0.102	0.217
PTH (pg/mL)	41.9 (31.9 - 53.3)	40.6 (32.0 - 51.9)	40.6 (31.0 - 52.1)	38.1 (30.8 - 49.6)	0.176
Beta-CTX (ng/L)	384.0 (277.0 - 488.0)	398.0 (293.5 - 542.0)	409.5 (290.3 - 540.8)	388.0 (274.0 - 546.0)	0.413
P1NP (g/L)	56.1 (43.0 - 71.2)	58.4 (45.1 - 76.4)	57.9 (44.1 - 75.1)	55.4 (41.9 - 72.8)	0.408

Supplementary Table 3. Differences in the clinical variables according to the serum levels of free 25OHD. Normally distributed variables are presented as means ±standard deviation, and non-normally distributed variables are presented as medians (interquartile range). The null hypothesis was tested using alpha = 0.05 (two-sided). ANOVA tests were used for normal data, and Kruskal-Wallis tests were used for non-normal data when multiple group comparisons were performed. [a]Adjusted for age, season and BMI.

Variables	rs2282679		rs12785878		rs10741657		rs6013897	
	**Beta**	***P* Value**	**Beta**	***P* Value**	**Beta**	***P* Value**	**Beta**	***P* Value**
**BMD**								
**Lumar 1-4**	0.0044	0.392	-0.0030	0.536	-0.0019	0.699	0.007958	0.225
**Femoral neck**	0.0019	0.678	-0.0046	0.176	-0.0009	0.799	0.003938	0.391
**Total hip**	0.0001	0.997	-0.0058	0.116	-0.0026	0.476	0.007057	0.152
**PTH**	0.0058	0.324	0.0060	0.270	0.0059	0.281	0.021064	0.004
**P1NP**	0.095	0.239	0.0027	0.756	-0.0053	0.545	-0.024969	0.030

Supplementary Table 4. Associations between the genetic IVs and the clinical variables that were observationally related to the total serum 25OHD levels. IVs, instrumental variables; Beta, regression coefficient. The False Discovery Rate (FDR) method was used to control the family-wise error rate when multiple hypotheses tests were performed. The null hypothesis was tested using alpha = 0.05 (two-sided). Significant values are presented in bold. The serum PTH and P1NP levels were log-transformed to approximate normality. Beta refers to the changes in the clinical traits per each additional copy of the minor allele. The analyses were adjusted for age, season and BMI.

**Potential confounding factors**	**rs2282679**		**rs12785878**		**rs10741657**		**rs6013897**	
	**Beta**	***P* Value**	**Beta**	***P* Value**	**Beta**	***P* Value**	**Beta**	***P* Value**
Age (years)	0.0317	0.921	0.1286	0.679	0.1540	0.621	1.3297	0.003
BMI (kg/m^2)	-0.0961	0.437	-0.0263	0.826	0.0026	0.983	-0.4222	0.008
Ca (mmol/L)	0.0054	0.126	0.0035	0.310	0.0041	0.225	-0.0099	0.032
P (mmol/L)	0.0013	0.805	-0.0013	0.800	-0.0074	0.139	-0.0100	0.133
Cr (μmol/L)	0.0739	0.879	0.1670	0.729	-0.5126	0.288	1.0009	0.126
BUN (mmol/L)	0.0056	0.929	-0.0078	0.898	-0.0369	0.545	0.0719	0.382

Supplementary Table 5. Associations between the genetic IVs and the potential confounding factors. IVs, instrumental variables; Beta, regression coefficient. Effect estimates are presented as changes in the potential confounding factor levels per each additional copy of the minor allele. The False Discovery Rate (FDR) method was used to control the family-wise error rate when multiple hypotheses tests were performed. The null hypothesis was tested using alpha = 0.05 (two-sided). Significant values are presented in bold.
